# Reduced IL-8 Secretion by NOD-like and Toll-like Receptors in Blood Cells from COVID-19 Patients

**DOI:** 10.3390/biomedicines11041078

**Published:** 2023-04-03

**Authors:** Laura E. Carreto-Binaghi, María Teresa Herrera, Silvia Guzmán-Beltrán, Esmeralda Juárez, Carmen Sarabia, Manuel G. Salgado-Cantú, Daniel Juarez-Carmona, Cristóbal Guadarrama-Pérez, Yolanda González

**Affiliations:** 1 Laboratorio de Inmunobiología de la Tuberculosis, Instituto Nacional de Enfermedades Respiratorias Ismael Cosío Villegas, Mexico City 14080, Mexico; 2 Departamento de Investigación en Microbiología, Instituto Nacional de Enfermedades Respiratorias Ismael Cosío Villegas, Mexico City 14080, Mexico; 3 Facultad de Medicina, Benemérita Universidad Autónoma de Puebla, Puebla 72000, Mexico; 4 Servicio de Urgencias, Instituto Nacional de Enfermedades Respiratorias Ismael Cosío Villegas, Mexico City 14080, Mexico

**Keywords:** COVID-19, agonist, TLRs function, NLRs function

## Abstract

Severe inflammatory responses are associated with the misbalance of innate and adaptive immunity. TLRs, NLRs, and cytokine receptors play an important role in pathogen sensing and intracellular control, which remains unclear in COVID-19. This study aimed to evaluate IL-8 production in blood cells from COVID-19 patients in a two-week follow-up evaluation. Blood samples were taken at admission (t1) and after 14 days of hospitalization (t2). The functionality of TLR2, TLR4, TLR7/8, TLR9, NOD1, and NOD2 innate receptors and IL-12 and IFN-γ cytokine receptors was evaluated by whole blood stimulation with specific synthetic receptor agonists through the quantification of IL-8, TNF-α, or IFN-γ. At admission, ligand-dependent IL-8 secretion was 6.4, 13, and 2.5 times lower for TLR2, TLR4, and endosomal TLR7/8 receptors, respectively, in patients than in healthy controls. Additionally, IL-12 receptor-induced IFN-γ secretion was lower in COVID-19 patients than in healthy subjects. We evaluated the same parameters after 14 days and observed significantly higher responses for TLR2, TLR4, TLR7/8, TLR9, and NOD1, NOD2, and IFN-γ receptors. In conclusion, the low secretion of IL-8 through stimulation with agonists of TLR2, TLR4, TLR7/8, TLR9, and NOD2 at t1 suggests their possible contribution to immunosuppression following hyperinflammation in COVID-19 disease.

## 1. Introduction

In December 2019, a novel coronavirus was reported in Wuhan, Hubei, China, causing several cases of atypical pneumonia [1]. During this pandemic, Mexico has been one of the most affected countries [2]. The distribution of COVID-19 cases in the Mexican population was similar in females and males. The most common comorbidities of this disease were hypertension, obesity, and diabetes mellitus [2].

Like SARS-CoV and MERS-CoV, the SARS-CoV-2 infection showed a hyperinflammatory state associated with COVID-19 pathogenesis, in which the innate immune system activation and lymphopenia are involved [3,4,5]. A meta-analysis reported increased neutrophil counts predictive for severity and mortality outcomes in COVID-19 patients [6]; moreover, neutrophil counts correlated to excessive oxidative stress [7]. The interaction of SARS-CoV-2 with the host cells induces the accumulation of reactive oxygen species (ROS), leading to progressive inflammation in severe COVID-19 [8]. Thiols (-SH-containing compounds) play an essential role in neutralizing free oxygen radicals, and glutathionylation of proteins increases during excessive ROS generation (reviewed in [9]). In COVID-19 patients with mild to moderate disease, low levels of thiol-disulfide have been reported [10], and the total thiol levels are inversely proportional to the severity of symptoms [11]. However, the imbalance among neutrophils and lymphocytes, its association with inflammation, and its evolution are unclear in COVID-19 patients.

Inflammation occurs in response to viral recognition by innate receptors [12]. Pathogen-associated molecular patterns (PAMPS), such as viral RNA, are released by infected epithelial cells. In silico analyses of SARS-CoV-2 mRNA interactions suggest an essential role of intracellular Toll-like receptors (TLRs) TLR3, TLR7, TLR8, and TLR9 in activating the inflammatory responses against the virus, mainly through the production of IL-8, TNF-α, and IL-1β via nuclear factor-κB (NF-κB) transcription activation [13]. In patients with COVID-19, neutrophils increase while lymphocytes decrease, thus modifying the N/L ratio [14]. Neutrophils express TLR and NOD-like receptors (NLR) in their cell surface; TLR2 and TLR4 recognize bacterial lipopeptides and lipopolysaccharides, whereas the intracellular TLR7 and TLR8 recognize viral single-stranded RNA, and TLR9 detects unmethylated CpG dinucleotides from bacterial DNA [15]. The NLR family includes cytosolic innate immune sensors NOD1 and NOD2; NOD1 recognizes γ-d-glutamyl-meso-diaminopimelic acid (iE-DAP), and NOD2 recognizes MurNAc-L-Ala-D-isoGln (MDP) [16]. TLR and NLR agonists induce IL-8 and other proinflammatory cytokines, via NF-kB-related pathways.

Cytokines modulate innate and adaptive immune responses. IL-12 mediates a broad range of effects on both innate and acquired immunity. IL-12 and IL-12 receptor (IL-12R) engagement induces IFN-γ production, which triggers T cell proliferation and stimulates NK and CD8 T cell responses against viruses [17]. IFN-γ signals through the IFN-γ receptor (IFN-γR), leading to the activation of JAK1, JAK2, and NF-kB to induce TNF-α release [18]. Type I and II interferons are essential and nonredundant for antiviral immune responses [19]. In mice, during SARS-CoV-2 infection, TNF-α and IFN-γ cause a lethal cytokine shock with tissue damage and exacerbated inflammation in COVID-19 [20].

The excessive inflammation induced by TLRs/NLRs ligation may cause tolerance and cross-tolerance to other innate receptor ligands, rendering the innate receptors refractory to subsequent pathogen recognition, leading to defective activation, chemotaxis, phagocytosis, and intracellular killing of other pathogens that result in susceptibility to opportunistic infections and pneumonia [21,22,23]. Hallmarks of the acute respiratory distress syndrome induced by MERS and SARS include a paradoxical hyperinflammation and immunosuppression state characterized by overactivation of the complement system, a lack of adequate adaptive immune responses, and an exaggerated innate immune response in predisposed individuals [24], yet the specific pathways are poorly understood. Since innate and adaptive responses are crucial to respond to SARS-CoV-2 infection, this study aimed at the assessment of the function of TLR and NOD agonists, as well as IL-12 and IFN-γ receptors, in a neutrophil-dominant cellular imbalance at the time of patient admission and two weeks later, when patient improvement was observed.

## 2. Materials and Methods

### 2.1. Study Group

We enrolled 30 hospitalized patients diagnosed with COVID-19 and 12 healthy subjects (without previous COVID-19 infection) at the National Institute for Respiratory Diseases (INER) in Mexico City, Mexico. All participants or their next of kin gave written informed consent for this study under the Declaration of Helsinki. The Institutional Review Board approved this study (C57-20). All patients received medication with steroids during their hospitalization, according to the international COVID-19 treatment guidelines developed from the RECOVERY trial [25]. We obtained ten milliliters of whole venous blood by standard phlebotomy at admission (t1) and two weeks after that (t2). Four milliliters of the whole blood were used for functional receptor evaluation, and from the centrifugation of six milliliters, we obtained the serum for total thiols determination.

### 2.2. Total Thiol Quantification

To assess the oxidation state, we quantified the total thiol serum levels using Cell Biolabs’ Total Thiol Assay Kit (San Diego, CA, USA), which includes a colorimetric probe that covalently reacts with the sulfhydryl group to release a chromophore; the absorbance of the plate was read at 450 nm. The thiol content in the samples was compared with a predetermined reduced glutathione standard curve. The assay was performed according to the manufacturer’s specifications, and the results were reported in µM. Reagent information is available in the Supplementary Table S1.

### 2.3. Innate Receptors Functionality

The functionality of innate TLRs and NLRs is crucial to sense pathogens and induce an immunologic response. Per well, we added 400 µL of whole blood diluted 1:1 with RPMI 1640 culture medium supplemented with 2 mM L-glutamine (Lonza, Walkersville, MD, USA) (RPMIc), in 24-well culture plates (Corning Costar Co., Corning, NY, USA). The diluted whole blood was stimulated with NLR ligands: NOD1-TriDAP (10 µg/mL) and NOD2-MDP (10 µg/mL), and TLR ligands: TLR4-LPS (100 ng/mL), TLR2-Pam-3-Cys (20 ng/mL), TLR7/8-Gardiquimod (5 µg/mL), and TLR9-CpGDNA (5 µg/mL) and incubated for 24 h at 37 °C and 5% CO_2_ atmosphere. The supernatants were harvested and stored at −70 °C until quantification of IL-8 production by ELISA, according to the manufacturer’s instructions of the reagent kit (Mabtech, Nacka Strand, Sweden); results were reported in pg/mL. Reagent information is available in the Supplementary Table S1.

### 2.4. Cytokine Receptors Functionality

Cytokine receptors of IFN-γ and IL-12 are essential in viral infections. For the evaluation of IL-12 receptor (IL-12R) function, 400 μL of the whole blood diluted 1:1 in RPMIc per well was stimulated with PMA (25 ng/mL), rhIL-12 (25 ng/mL), and PMA (25 ng/mL) plus rhIL-12 (25 ng/mL) and incubated for 24 h at 37 °C and 5% CO_2_ atmosphere; culture supernatants were harvested and stored at −70 °C until IFN-γ quantification by ELISA. Increases in the IFN-γ production after rhIL-12 plus PMA stimulation, relative to PMA alone, were expected. For IFN-γ receptor (IFN-γR), 400 μL of the whole blood diluted 1:1 in RPMI per well was stimulated with LPS (100 ng/mL) and LPS (100 ng/mL) plus rhIFN-γ (1000 IU/mL). After incubation for 24 h at 37 °C and 5% CO_2_ atmosphere, culture supernatants were harvested and stored at −70 °C until TNF-α quantification by ELISA; increases in TNF-α production after rhIFN-γ plus LPS stimulation, relative to LPS alone, were expected. IFN-γ and TNF-α production was measured by ELISA. Results were reported in pg/mL. Patients’ IL-12R and IFN-γR responses were defined in terms of the 95% CI of the healthy control median values. Reagent information is available in the Supplementary Table S1.

### 2.5. Statistical Analyses

Comparisons between COVID-19 patients and healthy controls were performed using the non-parametric Mann–Whitney’s U test, and the non-parametric Wilcoxon’s ranks sum test was used to analyze paired samples. Results are presented as medians and ranks. Categorical data are presented as numbers and percentages, and laboratory findings are presented as medians with an interquartile range (IQR). Statistical significance was considered with *p* ≤ 0.05. All statistical analyses were performed with GraphPad Prism version 9.3.1 for Mac (San Diego CA, USA).

## 3. Results

### 3.1. Study Groups

The characteristics of the patients were consistent with those reported for patients with the severe form of the disease. Most patients were male (76.6%) and presented with a severe disease (86.7%) that required mechanical ventilation during hospitalization. The median age was 52 years. The population was predominantly obese, with a median body mass index of 29.9. The most frequent comorbidities were obesity, hypertension, diabetes, smoking, and cardiac disease. COVID-19 patients had increased leukocyte and neutrophil counts and reduced lymphocyte counts. In addition, the patients had high serum glucose, fibrinogen, troponin A, ferritin, LDH, and C-reactive protein (Table 1). The healthy controls were selected because they had a negative COVID-19 test, and their laboratory findings were within the normal range.

### 3.2. Neutrophil/Lymphocyte Ratio in the COVID-19 Patients

**Figure 1:**
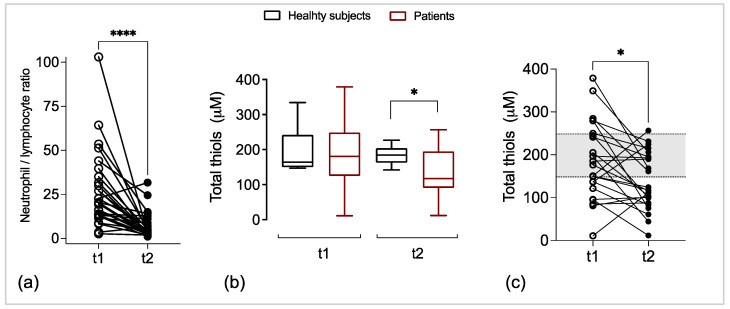
N/L ratio and thiol levels. (**a**) The N/L ratio before–after plots with the patients’ values at admission (t1) and two weeks later, during inpatient treatment (t2). (**b**) Total thiol levels in the sera of COVID-19 patients and healthy subjects at t1 and t2 using a commercial colorimetric assay. Box plots depict medians and quartiles. (**c**) Individual total thiol levels at t1 and t2; shaded areas flanked by dotted lines indicate the upper and lower limits of the 95% CI of the healthy subjects’ median thiol levels. * *p* < 0.05, **** *p* < 0.0001.

An increased neutrophil/lymphocyte (N/L) ratio and hyperinflammatory responses have been associated with COVID-19 [6,26]. We analyzed the N/L ratio in the COVID-19 patients at admission (t1) and after 14 days of inpatient treatment (t2). At t1, we found an N/L ratio of 20.4, meaning that the overall immune response of the patients was associated with a predominant neutrophil population [27]. However, at t2, we found a significant decrease in the N/L ratio to 3.8 (Figure 1a). The 5.4-fold N/L ratio reduction represents a recovery of the lymphocyte population.

### 3.3. Thiol-Disulfide Levels in the COVID-19 Patients

Low levels of thiol-disulfide and a correlation between high levels of ROS and the neutrophil count have been reported in COVID-19 patients [7], suggesting an oxidative imbalance state. Here, we analyzed the total thiol levels in patients at admission and at two-week follow-up during hospitalization (t2) to pursue the redox state. While total thiol levels were similar at t1 and t2 in the healthy subjects (thiols µM, median = t1: 164 vs. t2: 185), COVID-19 patients had a significant total thiols reduction at t2 (thiols µM, median = t1: 181 vs. t2: 117, Figure 1b). Only three patients recovered their thiol levels at t2 (Figure 1c). Half of the subjects exhibited thiol levels within the median 95% CI upper and lower limits of the healthy controls’ median (shaded area).

### 3.4. Functionality of Innate and Cytokine Immune Receptors in COVID-19 Patients at Admission

**Figure 2:**
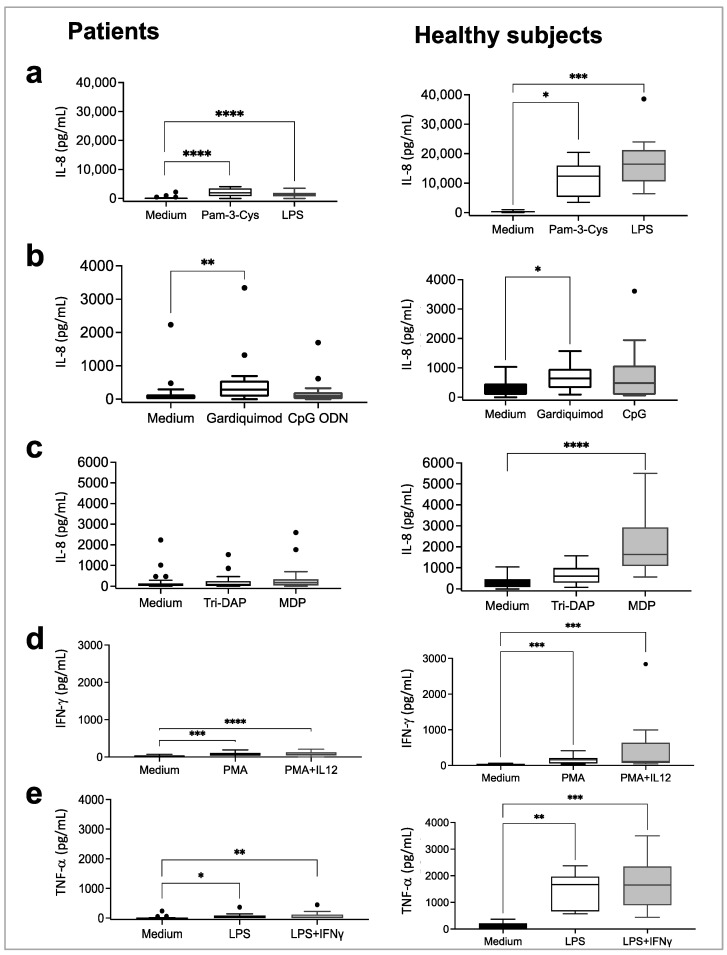
The functionality of innate and adaptive immune receptors in critically ill COVID-19 patients and healthy subjects at admission. Whole blood diluted 1:1 with culture medium was stimulated with specific ligands of innate immunity receptors (**a**–**c**): TLR2 (Pam-3-Cys, 2 µg/mL), TLR4 (LPS, 100 ng/mL), TLR7/8 (Gardiquimod, 5 µg/mL), TLR9 (CpG ODN, 10 µg/mL), NOD1 (Tri-DAP, 10 µg/mL), and NOD2 (MDP, 10 µg/mL), as well as specific ligands of cytokine receptors (**d**,**e**): IFN-γR (LPS, 100 ng/mL and rhIFN-γ, 1000 U/mL) and IL-12R (PMA, 25 ng/mL and rhIL-12, 25 ng/mL). Plasma levels of IL-8, TNF-α, and IFN-γ were measured as readout of ligand-dependent responses, by ELISA. Box plots indicate medians and quartiles. * *p* < 0.05, ** *p* < 0.01, *** *p* < 0.001, **** *p* < 0.0001.

The activation of most innate receptors by their cognate ligand triggers the production of IL-8 [28]. We used this feature to evaluate whether innate receptors were functional at admission (t1) compared with healthy subjects. IL-8 secretion at t1 and t2 after 24 h without stimulation from COVID19 patients is shown in Supplementary Table S2. We found a significant production of IL-8 after specific ligand stimulation of innate membrane receptors TLR2 and TLR4 (Pam-3-Cys and LPS, respectively) in patients. A similar response was observed in the healthy controls (Figure 2). Although the receptors responded to the TLR agonists, the observed response was lower in patients with COVID-19. In the patients, the stimulation of innate cell surface receptors with Pam-3-Cys resulted in a 6.4 times reduction in IL-8 secretion, while LPS stimulation ensued 13 times lower than in the healthy subjects (median, pg/mL: TLR2/Pam-3-Cys: 1951 vs. 12,422, TLR4/LPS: 1259 vs. 16,440, patients vs. healthy controls) (Figure 2a). Regarding the stimulation of innate endosomal receptors, Gardiquimod elicited 2.2 times lower and CpG 5.1 times lower IL-8 secretion in patients than in healthy subjects (median, pg/mL: TLR7/8/Gardiquimod: 285 vs. 642, and TLR9/CpG: 95 vs. 486, patients vs. healthy controls) (Figure 2b). Stimulating the innate cytoplasmic receptor NOD1 with Tri-DAP did not induce IL-8 secretion in either group. NOD2 stimulated with MDP induced 9.2 times lower IL-8 secretion in COVID-19 patients than in healthy subjects (median, pg/mL: MDP: 177 vs. 1634) (Figure 2c).

In addition, cytokine receptors from IL-12 and IFN-γ axes have been reported to play a role against viruses [29]. The stimulation of the IL-12R induced a significant IFN-γ secretion in both groups (Figure 2d). However, COVID-19 patients induced 1.7 times lower IFN-γ secretion with rhIL-12 plus PMA and 2.1 times lower with PMA stimulation alone than healthy subjects (IFN-γ pg/mL, median = PMA: 76 vs. 157, rhIL-12: 35 vs. 40, and rhIL-12+PMA: 65 vs. 110, patients vs. healthy controls) (Figure 2d). Because IFN-γ potentiates the effects of LPS [30], we stimulated the IFN-γR with IFN-γ plus LPS and measured the TNF-α secretion in COVID-19 patients and healthy controls. COVID-19 patients produced lower TNF-α, 62 times lower with LPS and 83 times lower with LPS + rhIFN-γ than healthy subjects (TNF-α pg/mL, median = LPS: 27 vs. 1671, and LPS + rhIFN-γ: 20 vs. 165, patients vs. healthy controls) (Figure 2e).

### 3.5. Follow-Up of the Immune Receptors’ Functionality in COVID-19 Patients

**Figure 3:**
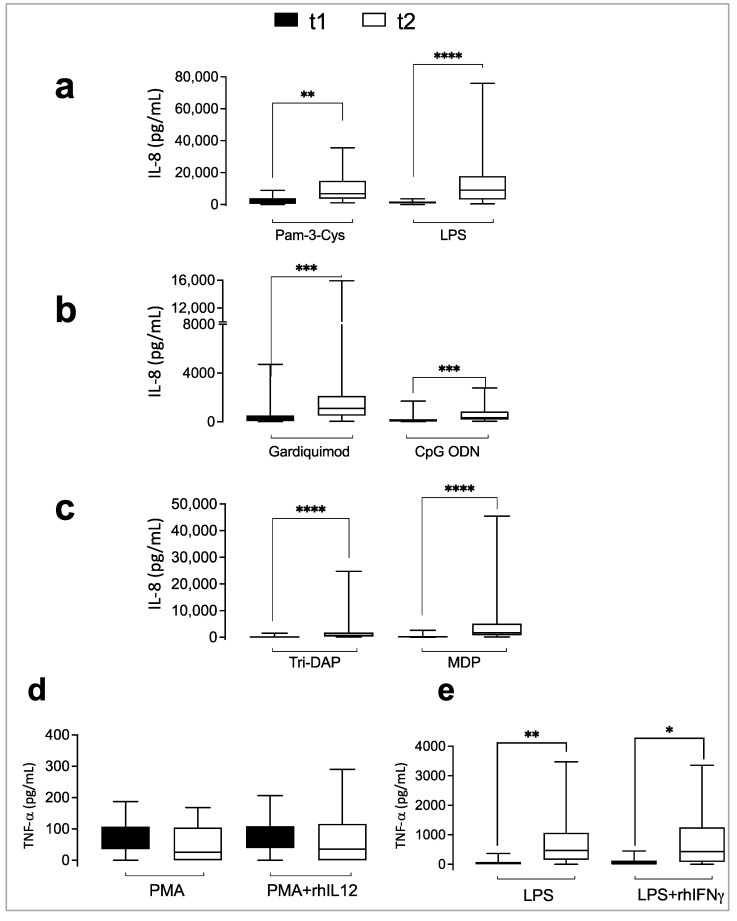
Immune receptors are functional in critically ill COVID-19 patients two weeks after admission. Whole blood diluted 1:1 with culture medium was stimulated with specific ligands of innate (**a**–**c**) and adaptive (**d**,**e**) immune receptors, as described in Materials and Methods. Depicted are box plots and pairwise comparisons between t1 (at admission) and t2 (two weeks later during hospitalization). * *p* < 0.05, ** *p* < 0.01, *** *p* < 0.001, **** *p* < 0.0001.

The response of innate receptors during hospitalization has yet to be investigated thoroughly. We compared the immune receptor functionality of critically ill COVID-19 patients at admission (t1) and after 14 days of hospitalization (t2). A significant increase in IL-8 levels was observed at t2 after agonist stimulation of TLR2, TLR4, TLR7/8, TLR9, NOD1, and NOD2 (Figure 2a–c and Figure 3a–c). We did not find a significant change in the IL-12R functionality (Figure 2d and Figure 3d); however, the cytokine receptor IFN-γR significantly increased TNF-α production at t2 after LPS or LPS plus rhIFN-γ stimulation (Figure 2e and Figure 3e), suggesting that blood immune cells from COVID-19 patients recovered their capacity to produce IL-8 and TNF-α after 14 days.

**Figure 4:**
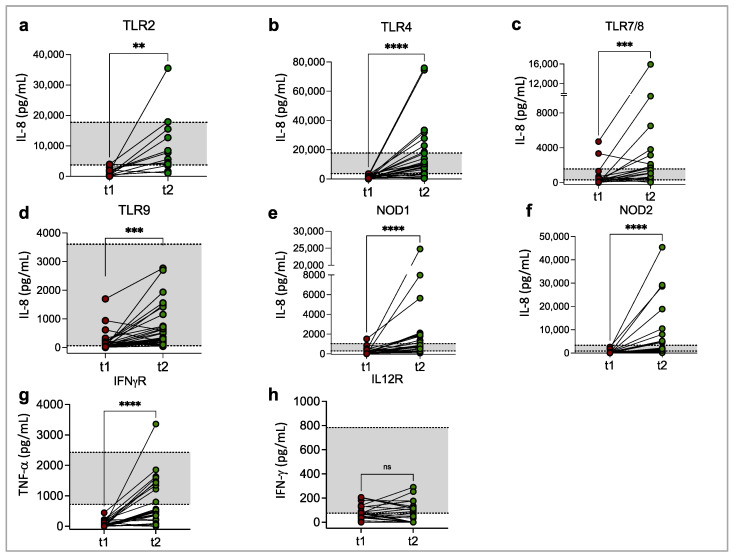
Individual analysis of the recovery of immune responses in COVID-19 patients. Before–after plots with the patients’ innate (**a**–**f**) and cytokine (**g**,**h**) responses at admission (t1) and two weeks later during hospitalization (t2) are depicted. Shaded areas flanked by dotted lines indicate the upper and lower limits of the 95% CI of the healthy controls’ median responses. ** *p* < 0.01, *** *p* < 0.001, **** *p* < 0.0001, ns = not significant.

Although, as a group, the patients recovered their ability to respond to agonist challenges, some remained unresponsive. We analyzed individual responses at t1 and t2 and observed the median 95% CI of the healthy controls (grey shaded areas in the plots of Figure 4 denote upper and lower limits). We found that most patients recovered TLR2, TLR4, TLR7/8, TLR9, NOD1, and NOD2 functionality (Figure 2 and Figure 4a–f). Regarding the cytokine receptors, while the IL-12R function remained low (Figure 2d and Figure 4g), half of the patients increased IFN-γ production after rhIFN-γR plus PMA stimulation at t2 (Figure 2e and Figure 4h).

### 3.6. Correlation between the N/L Ratio and TLRs, NLRs, and Cytokine Receptors in COVID-19 Patients

**Figure 5:**
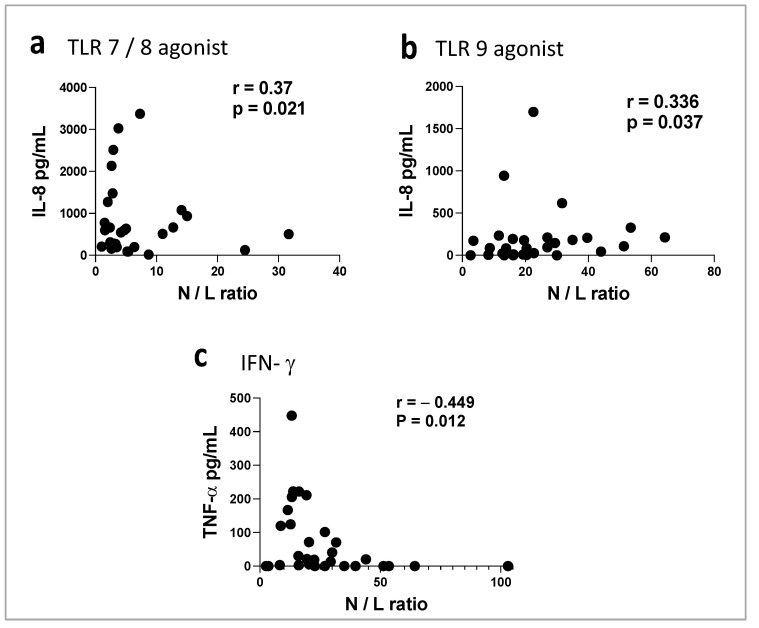
Correlation between cytokines production by agonist stimulation or IFN-γ stimulation vs. N/L ratio in COVID-19 patients. Dot plot of TLR7/8 and TLR9 receptor responses (secreting IL8) vs. N/L ratio (**a**,**b**), and IFN-γ receptor response (secreting TNF-α) vs. N/L ratio (**c**).

To identify whether innate and adaptive immune receptor dysfunction is due to an imbalance of neutrophil and lymphocyte counts at admission, we examined the correlation between the N/L ratio and the response of each innate or cytokine receptor. We found a moderate positive correlation between the N/L ratio and the dysfunction of TLR7/8 and TLR9 intracellular receptors (Figure 5a,b) and a moderate negative correlation between the N/L ratio and the dysfunction of IFN-γR (Figure 5c). The other receptors did not show a significant correlation. These data suggest that receptor dysfunction is due to an intrinsic cell response but not the cell count per se.

Other parameters, such as underlying infections, total lymphocyte count, and oxidative stress, could affect the response of the receptors. We compared subjects with dysfunctional receptors vs. subjects without dysfunctional receptors and we did not find a significant association between receptor dysfunction and the occurrence of healthcare-associated infections, total leukocyte counts, or total thiol levels, suggesting that receptor dysfunction is independent of other clinical factors.

## 4. Discussion

This study evaluated how the immune receptors’ response modified during SARS-CoV-2 infection after two weeks of inpatient care. We evaluated the function of innate and cytokine immune receptors and the N/L ratio and thiol production contribution to the IL-8-induction.

Even though the neutrophil/lymphocyte (N/L) ratio has been reported as a risk factor for COVID-19 [31], the biological mechanisms contributing to the IL-8 induction are not fully understood. The increased N/L ratio is due to an increase in the neutrophil count and a decrease in the lymphocyte count. Here, we found a high N/L ratio in critically ill patients with COVID-19 at admission; however, the N/L ratio decreased in most patients after two weeks of hospitalization, regardless of their clinical outcome. Despite this, IL-8 secretion increased, suggesting a mechanism independent of the number of neutrophils. Neutrophils are the primary ROS producers, and the balance between ROS and antioxidants such as thiols indicates oxidative stress. We found no difference between thiol levels in severe COVID-19 patients at admission and healthy subjects. Unlike patients with mild to moderate COVID-19 [10], severe COVID-19 patients had lower levels of thiol-disulfide after two weeks of inpatient care when neutrophil counts also decreased; thus, this reduction in thiols in critically ill patients may be associated with an increase in oxidative stress, since a high ROS production has been reported in ICU patients [32]. Moreover, the thiol-disulfide reduction in COVID-19 patients might be related to an enhanced inflammation via NF-κB activation [33]; however, only a weak correlation with Tri-DAP inducing IL-8 secretion and total thiols was observed, suggesting that the primary pathway is through receptor agonists. Further studies are required to elucidate the molecular pathways responsible for this phenomenon.

The activation of TLRs elicits antiviral and inflammatory responses during coronaviral infection [34]. However, to avoid hyperinflammation and consequent host tissue damage, the down-activation of TLR signaling by prolonged or repeated exposure to TLR ligands leads to no or low receptor response (tolerization) [35]. Tolerance is a protective mechanism that limits hyperinflammation and prevents septic shock and tissue damage. Here, we observed that, at admission, COVID-19 patients had a low response to the agonist of TLR2, TLR4, TLR7/8, and TLR9, suggesting a TLR cross-tolerance. SARS-CoV-2 components, such as the envelope protein recognized by TLR2, probably induce TLR tolerance [36]. In addition, COVID-19 patients had a lower response to the agonist of NOD2; this innate receptor has been previously reported to recognize viruses through their ssRNA [37], and NLRs and other members of the inflammasomes are activated during severe COVID-19 and in SARS-CoV-2-infected human monocytes [38]. In addition, in vitro studies showed that SARS-CoV-2 ORF3a, ORF7a, M, and N proteins induce inflammatory cytokine expression by activating NF-κB in HeLa cells [39]. Although tolerization has not been reported for NOD2, the inhibitory effects of Erbin are downstream after its ligand recognition, inhibiting NF-κB-dependent NOD2 and the inflammatory response [40]. The interaction between Erbin and viral proteins has been described for HIV and some coronaviruses [41]; however, the specific interaction with SARS-CoV-2 proteins with NOD-like receptors remains unclear.

The TNF-α and IFN-γ are related to cytokine storm-induced mortality in patients with COVID-19 [20]. We observed that COVID-19 patients had a lower response to the agonists of IL-12R and IFN-γR at admission, as previously reported in SARS-CoV and MERS infections, where this event diminishes T cell activation [24], probably related to cytokine receptor signaling.

Two weeks after admission, while COVID-19 patients were still hospitalized, we evaluated the innate and cytokine receptors’ response again, finding a recovery of their functionality in most of the patients, also associated with a decrease in the N/L ratio, despite the treatment with steroids. These findings are relevant to patients who did not recover their innate receptors’ functionality and might be at greater risk of defective recognition of subsequent viral or bacterial infections. However, the mechanisms are yet to be elucidated through further analyses.

The limitations of the present investigation include the sample size, lack of viral load assessment in COVID-19 patients, lack of moderate and mild COVID-19 patients to compare receptor responses, and SARS-CoV2 molecular patterns specific receptor responses.

In conclusion, low secretion of IL-8 was observed upon stimulation with TLR2, TLR4, TLR7/8, TLR9, and NOD2 agonists by the blood cells of COVID-19 patients at hospital admission and was restored after two weeks of hospitalization, which may be a contributing factor to immunosuppression.

## Figures and Tables

**Table 1:** Baseline characteristics of the studied population.

Characteristics	Patients, n = 30	Normal Range
Male, n (%)	23 (76.6%)	
Age, median (IQR)	52 (10.5)	
Body Mass Index, median (IQR)	29.9 (6.2)	18.5–24.9
Deaths, n (%)	10 (33.3%)	
Chronic comorbidities		
Diabetes, n (%)	5 (16.7%)	
Hypertension, n (%)	9 (30%)	
Chronic cough, n (%)	1 (3.3%)	
Cardiac disease *, n (%)	2 (6.7%)	
Lung disease **, n (%)	1 (3.7%)	
Obesity, n (%)	15 (50%)	
Alcoholism, n (%)	1 (3.3%)	
Smoking	3 (10%)	
Others ***, n (%)	7 (23.3%)	
Laboratory findings		
Cell blood counts		
Hematocrit (%), median (IQR)	46.6 (4.9)	43.5–52.5
Hemoglobin (g/dL), median (IQR)	16 (1.7)	14.5–17.5
Leucocytes (103/mm^3^), median (IQR)	12.5 (5.4)	4.5–11.0
Neutrophils (103/mm^3^), median (IQR)	10.8 (5.15)	1.8–7.7
Lymphocytes (103/mm^3^), median (IQR)	0.5 (0.5)	1.0–4.8
Monocytes (103/mm^3^), median (IQR)	0.4 (0.2)	0–0.8
Eosinophils (103/mm^3^), median (IQR)	0 (0)	0.02–0.45
Basophils (103/mm^3^), median (IQR)	0 (0)	0.02–0.1
Platelets, median (IQR)	223,000 (106,250)	140,000–400,000
Serum levels		
Glucose (mg/dL), median (IQR)	140 (55)	74–118
Creatinine (mg/dL), median (IQR)	0.83 (0.28)	0.7–1.2
CPK (IU/L), median (IQR)	113.5 (199.7)	38–234
D dimer (µg/mL), median (IQR)	0.95 (5.42)	<0.5
PT (s), median (IQR)	15.75 (1.67)	12.8–17.4
PTT (s), median (IQR)	38.1 (10.72)	30–44
Fibrinogen (mg/dL), median (IQR)	696 (143.5)	238–498
High Sensitivity Troponin A (pg/mL), median (IQR)	21.1 (97)	Women: 13.8–17.5
		Men: 28.9–39.9
Ferritin (ng/mL), median (IQR)	978.5 (1424.20)	20–250
Blood Natriuretic Peptide (pg/mL), median (IQR)	47.15 (81.05)	<125
LDH (IU/L), median (IQR)	550 (231)	98–192
Alkaline phosphatase (IU/L), median (IQR)	90 (46.5)	38–126
C-reactive protein (mg/L), median (IQR)	13.48 (11.95)	<1
Procalcitonin (ng/L), median (IQR)	0.235 (0.38)	<0.5
Severity		
Mechanical ventilation at hospitalization, n (%)	26 (86.7%)	
Shock Index, median (IQR)	0.79 (0.28)	0.5–0.7

CPK: creatine phosphokinase; PT: prothrombin time; PTT: partial thromboplastin time; LDH: lactate dehydrogenase. * One patient had heart failure, and the other had angina pectoris. ** One patient had Chronic Obstructive Pulmonary Disease. *** Two patients presented with obstructive sleep apnea, three with allergic diseases, one with diabetic neuropathy, and one with sinusitis.

## Data Availability

The data presented in this study are available on request from the corresponding author. The data are not publicly available due to the privacy of the participants.

## Supplementary Materials

The following supporting information can be downloaded at: https://www.mdpi.com/article/10.3390/biomedicines11041078/s1, Table S1: Reagents list. Table S2: IL-8 secretion after 24 h whole blood culture from COVID-19 patients.

## References

[1] Huang C., Wang Y., Li X., Ren L., Zhao J., Hu Y., Zhang L., Fan G., Xu J., Gu X. (2020). Clinical features of patients infected with 2019 novel coronavirus in Wuhan, China. Lancet.

[2] Secretaría de Salud Dirección General de Epidemiología. COVID-19 México. https://datos.covid-19.conacyt.mx/.

[3] Mortaz E., Tabarsi P., Varahram M., Folkerts G., Adcock I.M. (2020). The Immune Response and Immunopathology of COVID-19. Front. Immunol..

[4] Wiersinga W.J., Rhodes A., Cheng A.C., Peacock S.J., Prescott H.C. (2020). Pathophysiology, Transmission, Diagnosis, and Treatment of Coronavirus Disease 2019 (COVID-19): A Review. JAMA.

[5] Chen G., Wu D., Guo W., Cao Y., Huang D., Wang H., Wang T., Zhang X., Chen H., Yu H. (2020). Clinical and immunological features of severe and moderate coronavirus disease 2019. J. Clin. Investig..

[6] Li X., Liu C., Mao Z., Xiao M., Wang L., Qi S., Zhou F. (2020). Predictive values of neutrophil-to-lymphocyte ratio on disease severity and mortality in COVID-19 patients: A systematic review and meta-analysis. Crit. Care.

[7] Veenith T., Martin H., Le Breuilly M., Whitehouse T., Gao-Smith F., Duggal N., Lord J.M., Mian R., Sarphie D., Moss P. (2022). High generation of reactive oxygen species from neutrophils in patients with severe COVID-19. Sci. Rep..

[8] Beltrán-García J., Osca-Verdegal R., Pallardó F.V., Ferreres J., Rodríguez M., Mulet S., Sanchis-Gomar F., Carbonell N., García-Giménez J.L. (2020). Oxidative stress and inflammation in COVID-19-associated sepsis: The potential role of anti-oxidant therapy in avoiding disease progression. Antioxidants.

[9] Baba S.P., Bhatnagar A. (2018). Role of thiols in oxidative stress. Curr. Opin. Toxicol..

[10] Dagcioglu B.F., Keskin A., Guner R., Kaya Kalem A., Eser F., Erel O., Neselioglu S., Bayrakdar F., Ozkara A. (2021). Thiol levels in mild or moderate COVID-19 patients: A comparison of variant and classic COVID-19 cases. Int. J. Clin. Pract..

[11] Kalem A.K., Kayaaslan B., Neselioglu S., Eser F., Hasanoglu İ., Aypak A., Akinci E., Akca H.N., Erel O., Guner R. (2021). A useful and sensitive marker in the prediction of COVID-19 and disease severity: Thiol. Free Radic. Biol. Med..

[12] Mehta P., McAuley D.F., Brown M., Sanchez E., Tattersall R.S., Manson J.J. (2020). COVID-19: Consider cytokine storm syndromes and immunosuppression. Lancet.

[13] Choudhury A., Das N.C., Patra R., Mukherjee S. (2021). In silico analyses on the comparative sensing of SARS-CoV-2 mRNA by the intracellular TLRs of humans. J. Med. Virol..

[14] Jimeno S., Ventura P.S., Castellano J.M., García-Adasme S.I., Miranda M., Touza P., Lllana I., López-Escobar A. (2021). Prognostic implications of neutrophil-lymphocyte ratio in COVID-19. Eur. J. Clin. Investig..

[15] Prince L.R., Whyte M.K., Sabroe I., Parker L.C. (2011). The role of TLRs in neutrophil activation. Curr. Opin. Pharmacol..

[16] Ekman A.K., Cardell L.O. (2010). The expression and function of Nod-like receptors in neutrophils. Immunology.

[17] Komastu T., Ireland D.D., Reiss C.S. (1998). IL-12 and viral infections. Cytokine Growth Factor Rev..

[18] Lee A.J., Ashkar A.A. (2018). The Dual Nature of Type I and Type II Interferons. Front. Immunol..

[19] Müller U., Steinhoff U., Reis L.F., Hemmi S., Pavlovic J., Zinkernagel R.M., Aguet M. (1994). Functional role of type I and type II interferons in antiviral defense. Science.

[20] Karki R., Sharma B.R., Tuladhar S., Williams E.P., Zalduondo L., Samir P., Zheng M., Sundaram B., Banoth B., Malireddi R.K.S. (2021). Synergism of TNF-α and IFN-γ Triggers Inflammatory Cell Death, Tissue Damage, and Mortality in SARS-CoV-2 Infection and Cytokine Shock Syndromes. Cell.

[21] Kumar V. (2020). Pulmonary Innate Immune Response Determines the Outcome of Inflammation During Pneumonia and Sepsis-Associated Acute Lung Injury. Front. Immunol..

[22] Famà A., Midiri A., Mancuso G., Biondo C., Lentini G., Galbo R., Giardina M.M., De Gaetano G.V., Romeo L., Teti G. (2020). Nucleic acid-sensing toll-like receptors play a dominant role in innate immune recognition of pneumococci. mBio.

[23] Carty M., Bowie A.G. (2010). Recent insights into the role of Toll-like receptors in viral infection. Clin. Exp. Immunol..

[24] Jacques F.H., Apedaile E. (2020). Immunopathogenesis of COVID-19: Summary and Possible Interventions. Front. Immunol..

[25] Horby P., Lim W.S., Emberson J.R., Mafham M., Bell J.L., Linsell L., Staplin N., Brightling C., Ustianowski A., RECOVERY Collaborative Group (2021). Dexamethasone in Hospitalized Patients with COVID-19. N. Engl. J. Med.

[26] Liu Y., Du X., Chen J., Jin Y., Peng L., Wang H.H.X., Luo M., Chen L., Zhao Y. (2020). Neutrophil-to-lymphocyte ratio as an independent risk factor for mortality in hospitalized patients with COVID-19. J. Infect..

[27] Qun S., Wang Y., Chen J., Huang X., Guo H., Lu Z., Wang J., Zheng C., Ma Y., Zhu Y. (2020). Neutrophil-to-Lymphocyte Ratios Are Closely Associated With the Severity and Course of Non-mild COVID-19. Front. Immunol..

[28] Fritz J.H., Girardin S.E., Fitting C., Werts C., Mengin-Lecreulx D., Caroff M., Cavaillon J.M., Philpott D.J., Adib-Conquy M. (2005). Synergistic stimulation of human monocytes and den-dritic cells by Toll-like receptor 4 and NOD1- and NOD2- activating agonists. Eur. J. Immunol..

[29] Novelli F., Casanova J.L. (2004). The role of IL-12, IL-23 and IFN-gamma in immunity to viruses. Cytokine Growth Factor Rev..

[30] Lee J.Y., Sullivan K.E. (2001). Gamma interferon and lipopolysaccharide interact at the level of transcription to induce tumor necrosis factor alpha expression. Infect. Immun..

[31] Yang A.P., Liu J.P., Tao W.Q., Li H.M. (2020). The diagnostic and predictive role of NLR, d-NLR and PLR in COVID-19 patients. Int. Immunopharmacol..

[32] Ayala J.C., Grismaldo A., Sequeda-Castañeda L.G., Aristizábal-Pachón A.F., Morales L. (2021). Oxidative Stress in ICU Patients: ROS as Mortality Long-Term Predictor. Antioxidants.

[33] Kabe Y., Ando K., Hirao S., Yoshida M., Handa H. (2005). Redox regulation of NF-kappaB activation: Distinct redox regulation between the cytoplasm and the nucleus. Antioxid. Redox Signal..

[34] Dyavar S.R., Singh R., Emani R., Pawar G.P., Chaudhari V.D., Podany A.T., Avedissian S.N., Fletcher C.V., Salunke D.B. (2021). Role of toll-like receptor 7/8 pathways in regulation of interferon response and inflammatory mediators during SARS-CoV2 infection and potential therapeutic options. Biomed. Pharmacother..

[35] Butcher S.K., O’Carroll C.E., Wells C.A., Carmody R.J. (2018). Toll-Like Receptors Drive Specific Patterns of Tolerance and Training on Restimulation of Macrophages. Front. Immunol..

[36] Zheng M., Karki R., Williams E.P., Yang D., Fitzpatrick E., Vogel P., Jonsson C.B., Kanneganti T.D. (2021). TLR2 senses the SARS-CoV-2 envelope protein to produce inflammatory cytokines. Nat. Immunol..

[37] Godkowicz M., Druszczyńska M. (2022). NOD1, NOD2, and NLRC5 Receptors in Antiviral and Antimycobacterial Immunity. Vaccines.

[38] Wong L.Y.R., Perlman S. (2022). Immune dysregulation and immunopathology induced by SARS-CoV-2 and related coronaviruses—Are we our own worst enemy?. Nat. Rev. Immunol..

[39] Su C.M., Wang L., Yoo D. (2021). Activation of NF-κB and induction of proinflammatory cytokine expressions mediated by ORF7a protein of SARS-CoV-2. Sci. Rep..

[40] McDonald C., Chen F.F., Ollendorff V., Ogura Y., Marchetto S., Lécine P., Borg J.P., Nuñez G. (2005). A role for Erbin in the regulation of Nod2-dependent NF-kappaB signaling. J. Biol. Chem..

[41] Ivarsson Y., Arnold R., McLaughlin M., Nim S., Joshi R., Ray D., Liu B., Teyra J., Pawson T., Moffat J. (2014). Large-scale interaction profiling of PDZ domains through proteomic peptide-phage display using human and viral phage peptidomes. Proc. Natl. Acad. Sci. USA.

