# Supplementary material for: Reduced IL-8 Secretion by NOD-like and Toll-like Receptors in Blood Cells from COVID-19 Patients
Source: Biomedicines. 2023 Apr 3;11(4):1078. doi: 10.3390/biomedicines11041078 (PMC10136206; doi:10.3390/biomedicines11041078)
Supplement: Supplementary file 1 [file biomedicines-11-01078-s001.zip › biomedicines-2290164-supplementary.pdf]

Supplementary Materials

Reduced IL-8 Secretion by NOD-Like and Toll-Like Receptors in Blood Cells from COVID-19 Patients

Laura E. Carreto-Binaghi, María Teresa Herrera, Silvia Guzmán-Beltrán, Esmeralda Juárez, Carmen Sarabia, Manuel G. Salgado-Cantú, Daniel Juarez-Carmona, Cristóbal Guadarrama-Pérez and Yolanda González

Table S1. Reagents list.

Reagents	Source	Catalog
Total Thiol Assay Kit	Cell Biolabs	MET-5053
Tri-DAP	Invivogen	tlrl-tdap
MDP	Invivogen	tlrl-mdp
LPS	Sigma-Aldrich Co.	L-4391
Pam-3-Cys SKKKK	EMC microcollection	L2000
Gardiquimod	Invivogen	tlrl-gdq-5
Cp GDNA	Invivogen	tlrl-2395-1
IL-8 ELISA determination kit	Mabtech	3560-1A-20
PMA	Sigma-Aldrich Co.	79346
rhIL-12	R&D Systems	219-IL-025
rhIFN-γ	R&D Systems	285-IF-100
**TNF-**a **quantification by ELISA**		
1-TNF-a monoclonal Antibody	1-Invitrogen	Cat. M303
2-TNF-a monoclonal Antibody-Biotin	2-Invitrogen	Cat. M302B
3-Alkaline phosphatase-conjugated-streptavidin	3-Jakson ImmunoResearch	Cat.016-050-084
**IFN-g quantification by ELISA**		
1-Human IFN-g Mab,	1-Thermo Scientific	Cat. M700A
2-Human IFN-g Mab biotin labeled	2-Thermo Scientific	Cat.M701B
3-Alkaline phosphatase-conjugated streptavidin	3-Jakson ImmunoResearch	Cat.016-050-084

Table S2. IL-8 secretion after 24h whole blood culture from COVID-19 patients.

	**IL-8 (pg/ml)**	
	**t1**	**t2**
Without stimulation	78 [0-2234]	586 [0-3287]

t1=At admission, **t2**= two weeks later during inpatient treatment.
